# Tribological Performance of Grease-Coated Rubber in High-Pressure Hydrogen Storage Applications

**DOI:** 10.3390/polym18020284

**Published:** 2026-01-21

**Authors:** Sheng Ye, Haijie Zhi, Wenqiang Wu, Sohail Yasin, Chaohua Gu, Jianfeng Shi, Sheng Zeng

**Affiliations:** 1 Institute of Advanced Equipment, College of Energy Engineering, Zhejiang University, Hangzhou 310027, China; zjusheng@zju.edu.cn (S.Y.); 22427029@zju.edu.cn (H.Z.); guchaohua@zju.edu.cn (C.G.); shijianfeng@zju.edu.cn (J.S.); 2 Sichuan Dowhon New Materials Co., Ltd., Meishan 620000, China; 3 Hydrogen Energy Research Institute, Zhejiang University, Hangzhou 310027, China

**Keywords:** high-pressure hydrogen, nitrile butadiene rubber (NBR), grease coating, coefficient of friction, wear morphology

## Abstract

Rubber materials undergo continuous wear in high-pressure seal applications. To address the risk of adhesive wear and consequent leakage of rubber seals operating under reciprocating sliding in high-pressure hydrogen storage and refueling systems, this study employed high-pressure hydrogen tribology testing. Ball-on-disk reciprocating tests were conducted using a 316L stainless-steel ball against silica-filled nitrile butadiene rubber (NBR), and the friction response and wear-morphology evolution were compared under ambient air, 1 MPa hydrogen (H_2_), 50 MPa H_2_, 50 MPa nitrogen (N_2_), and grease-coated conditions. Under dry sliding, the coefficient of friction (COF) of NBR in air and hydrogen ranged from 1.34 to 1.44, whereas it decreased markedly to 0.942 in 50 MPa N_2_. The wear volume under the four dry conditions was concentrated in the range of ~0.292–0.320 mm^3^. After grease coating, the steady-state COF in air and at 50 MPa H_2_ dropped to 0.099 and 0.105, respectively, and the wear features changed from ridge-like wear patterns/tear pits to regular, smooth indentations with slight running marks. The results demonstrate that a lubricating film can effectively separate direct metal–rubber contact and suppress stick–slip, enabling a low-friction, low-wear, and highly stable interface in high-pressure hydrogen, and providing a practical engineering route for reliable operation of rubber seals in hydrogen service.

## 1. Introduction

Polymeric materials, owing to their good elasticity, corrosion resistance, and relatively low cost, are widely used as sealing components in high-pressure hydrogen systems [1]. Nevertheless, seals in such systems face complex friction and wear challenges. Under dynamic sealing, high-frequency reciprocating motion of compressor pistons, relative motion between valve spool/stem and valve body, and frequent coupling/decoupling of refueling nozzles can all induce wear. Under static sealing, pressure cycling may cause small reciprocating micro-slips of static seals, which can also lead to wear. Moreover, long-term exposure to extreme conditions such as high pressure and wide temperature ranges may trigger hydrogen uptake and swelling, blistering (bubble) damage, and degradation of mechanical properties in polymers [2,3,4]. The synergistic effects of these factors with friction and wear further increase the risk of seal failure. Industry surveys in the United States and Japan indicate that hydrogen leakage is among the major types of incidents in refueling stations [5]. Therefore, systematic investigation of the tribological behavior of sealing materials in high-pressure hydrogen is of considerable scientific and engineering value for reducing leakage risk, ensuring the safe operation of hydrogen equipment, and supporting the healthy development of the hydrogen industry.

Given the lack of data for polymers in hydrogen service, studies have been carried out to fill this gap in several key directions, including tribology, failure analysis, evolution of mechanical properties, and gas permeability [6]. In particular, the tribological behavior of polymers under hydrogen exposure has attracted growing attention in recent years. This interest stems from the extensive use of polymers in dry-sliding components of hydrogen applications, where friction and wear directly determine system reliability and long-term durability.

Recent experimental studies have begun to systematically elucidate the influence of hydrogen environments on friction and wear. It has been reported that the choice of nanofillers can affect tribological performance more strongly than the choice of polymer matrix [7]. However, replacing traditional nanofillers (carbon black, silica) with modern alternative fillers can complicate polymer processing [2], especially for rubber materials [8,9]. Indeed, filler modification and addition can improve rubber performance [10,11,12,13,14], but they also increase cost and engineering complexity. In high-pressure conditions relevant to practical infrastructure, nitrile butadiene rubber (NBR) elastomers showed higher friction in 26.2 MPa hydrogen than in argon or air, which was attributed to a combination of mechanisms including hydrogen-induced “pseudo-plasticization” (softening) and the formation of cavities at the filler–matrix interface [15]. Long-term wear driven by hydrogen permeation has also been highlighted as a major challenge for maintaining seal integrity [16]. It has been reported that applying an organic/inorganic coating on rubber can reduce the coefficient of friction (COF) at room temperature from 0.854 to 0.116, and after hydrogen exposure from 0.929 to 0.151 [17]. Similarly, incorporation of additives and fillers can lower COF and enhance rubber wear resistance in hydrogen [7,18]. Nevertheless, such formulated coatings and additives increase processing cost, and surface coatings may pose contamination risks to hydrogen purity. Although these studies are valuable, the overall literature remains limited. More systematic and comprehensive studies are still required, including comparisons among practically available rubber systems and mechanistic clarification of how specific additives or coatings regulate tribological performance in high-pressure hydrogen.

Herein, a pragmatic and feasible approach is applied: coating rubber surfaces with hydrogen-compatible grease. Specifically, a commercially available polytetrafluoroethylene (PTFE)-based grease was applied to the rubber surface, mimicking common industrial lubrication practice. This grease was selected for its chemical inertness to demonstrate the feasibility of the proposed approach. The objective was to examine the effect of the grease coating on the wear resistance and COF of rubber in high-pressure hydrogen. The results show that the PTFE-based grease coating reduces both COF and wear rate, providing a viable route to improve tribological performance and service life of rubber seals under hydrogen conditions.

## 2. Materials and Methods

### 2.1. Rubber Sample Material and Preparation

Rubber sealing materials commonly used in high-pressure hydrogen systems include NBR, hydrogenated nitrile butadiene rubber (HNBR), ethylene propylene diene monomer (EPDM), fluoroelastomer (FKM), and silicone rubber (VMQ). Among them, NBR is a copolymer of acrylonitrile and butadiene [19,20]. The acrylonitrile groups in its molecular chain impart excellent oil resistance, chemical resistance, and low gas permeability, while maintaining good mechanical strength and processability. NBR is widely used for static and dynamic sealing in the chemical, automotive, and aerospace industries, and is also one of the common sealing materials in high-pressure hydrogen systems [1,3].

Raw (uncured) NBR has limitations such as low strength, poor elasticity, and susceptibility to aging due to weak intermolecular interactions. Therefore, compounding formulations and vulcanization processes are required to obtain satisfactory properties. Vulcanization forms crosslink networks between rubber molecular chains, providing stable mechanical performance; fillers such as carbon black and silica further enhance strength, wear resistance, and processability, while reducing cost [21,22].

In this study, silica-filled NBR was selected as the sample material because hydrogen does not adsorb on silica. The base rubber was NBR 4155 with an acrylonitrile content of 41%. The silica filler was hydrophobic fumed silica (R974). A sulfur vulcanization system was used, together with activators, accelerators, and antioxidants. An internal mixer (HOOK, Shanghai Kechuang Co. Ltd., Shanghai, China) was employed to prepare the compounds. The NBR and additives listed in Table 1 were mixed at 100 °C and a rotor speed of 40 rpm for approximately 25 min. Vulcanized sheets were produced by compressing the mixture at 15 MPa using a vulcanizer (XL-25, Huzhou Xinli Rubber Machinery Co., Ltd., Huzhou, China) at 150 ± 5 °C for 30 min to a thickness of ~4.0 mm. The specimens, featuring a smooth molded surface, were cleaned with deionized water, wiped with clean non-woven cloth, and oven-dried at 40 °C for 8 h. Finally, they were stored in a dry box at room temperature (relative humidity ~25%) prior to testing. The main formulation is summarized in Table 1.

### 2.2. Friction and Wear Testing Functionality

**Figure 1:**
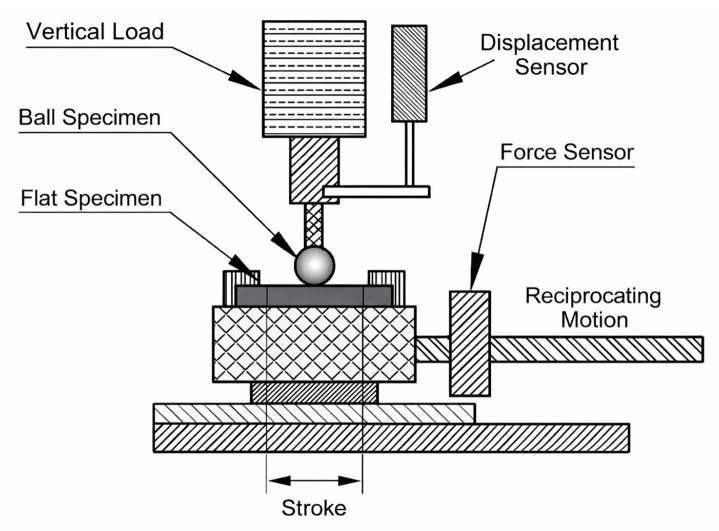
Schematic of a reciprocating ball-on-disk tribometer.

To address the dearth of tribometers capable of operating in high-pressure hydrogen (typically ≥35 MPa), we developed a custom-built in situ testing system. Unlike existing commercial devices, this rig achieves a maximum design pressure of 140 MPa and a temperature control range of −60 °C to 200 °C, covering the requirements of major hydrogen infrastructure use. The apparatus employs a linear reciprocating ball-on-disk configuration, with reference to the CSA/ANSI CHMC 2 standard (Figure 1).

**Figure 2:**
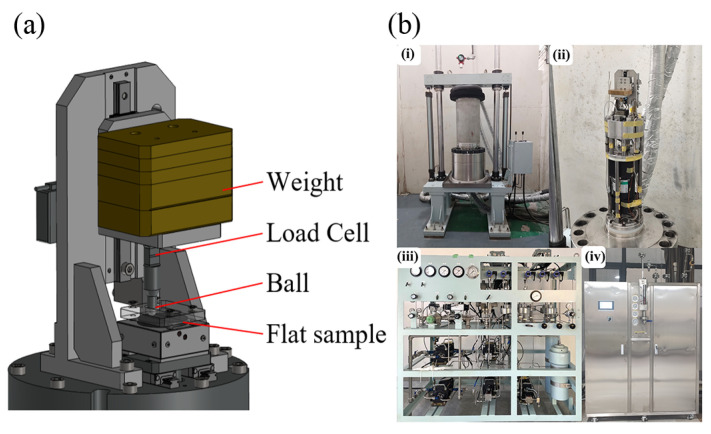
(**a**) In situ tribometer structure; (**b**) photograph of the system: (**i**) high-pressure hydrogen chamber, (**ii**) tribometer, (**iii**) integrated purging and gas-supply unit, and (**iv**) cooling/heating unit.

As shown in Figure 2a, the friction tester is placed inside the high-pressure hydrogen chamber. The reciprocating motion is driven by a motor coupled with a custom-designed cam mechanism, which converts rotary motion into linear motion with a constant velocity profile over the majority of the stroke. The normal load is applied directly using dead weights to ensure a constant force application. The friction force is measured in situ by a load cell utilizing strain gauges on a deformable element arranged in a bridge circuit.

The system integrates high-pressure gas supply, purging, and safety interlocks (Figure 2b). Internal pressure and temperature sensors are installed within the chamber to monitor the test environment in real time. Key technical specifications are summarized in Table 2.

### 2.3. High-Pressure Friction and Wear Testing and Characterization

Ball-on-disk reciprocating tests were conducted using the high-pressure hydrogen tribology testing, which provides a maximum pressure of 140 MPa and a temperature-control range of −60 to 200 °C. The rig can operate under controlled atmospheres such as air, hydrogen, or nitrogen. Both hydrogen and nitrogen were high-purity gases (99.999%). All tests were performed at room temperature (20 °C ± 5 °C). For tests in the air, the relative humidity was 40–60%. The ball specimen was a 316L stainless-steel ball with a diameter of 5.0 mm (grade G200, surface roughness Ra ≈ 0.15 μm). The NBR specimen had a diameter of 30 mm and a thickness of 4 mm. The tests followed CSA/ANSI CHMC 2, with basic parameters: normal load of 8.2 N, stroke length of 10 mm (20 mm per cycle), sliding speed of 1 mm/s, and 120 cycles. Comparative tests were performed in ambient air, 1 MPa H_2_, 50 MPa H_2_, and 50 MPa N_2_; additionally, a grease-coated condition was introduced to evaluate the regulation of interfacial behavior by a lubricating film. For lubricated tests, the PTFE-based grease (HSE-205-Shenzhen Haisheng Lubrication Technology Co. Ltd., Shenzhen, China) was uniformly spread on the NBR surface using a scraper to form a continuous thin film prior to mounting. Based on the worked penetration of the grease being 265–295 (0.1 mm) as per GB/T 269, it is classified as NLGI Grade 2. All other test parameters under lubricated conditions were kept identical to those under dry sliding. The counterface ball was not coated. The tests were conducted in situ within the high-pressure chamber. After mounting the specimens, the chamber was evacuated to ~0.85 MPa and purged twice with 1 MPa high-purity gas (H_2_ or N_2_). The chamber was then pressurized to the target pressure at a rate of ~2 MPa/min. The specimens were soaked at the test pressure for 6 h to ensure gas saturation before the friction test commenced. Each test condition was repeated three times. The detailed test matrix and conditioning protocols are summarized in Table 3.

**Figure 3:**
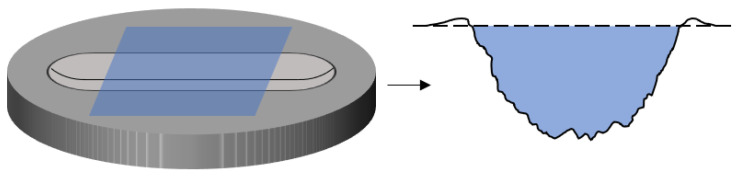
Average profile of the wear scar: wear volume corresponds to the blue area.

During testing, the friction force was continuously recorded to obtain friction–cycle curves, and the steady-state COF was calculated as the average over the last 10 cycles. Specifically, the COF for each cycle was calculated by averaging the absolute values of the friction force in the stable sliding regions of the forward and backward strokes, divided by the normal load of 8.2 N. Three-dimensional wear topographies and wear volumes were measured using a 3D optical profilometer at least 48 h after testing to allow for elastic recovery of the rubber. The wear scar width was determined by averaging measurements from five cross-sections uniformly selected along the wear track perpendicular to the sliding direction. The wear volume was calculated as the product of the stroke length and the cross-sectional area of the average profile extracted from the central region of the wear track (avoiding the reversal points at both ends). As illustrated in Figure 3, a straight reference line was drawn between the unworn areas on both sides of the wear scar, and the area of the groove below this line was used to compute the volume. An optical microscope and a field-emission scanning electron microscope (FE-SEM) were used to observe micro-morphologies before and after testing, and ATR-FTIR spectroscopy was used to screen for chemical-structure changes in the material.

## 3. Results

### 3.1. In Situ Friction and Wear Behavior Under Different Atmospheres and Pressures

**Figure 4:**
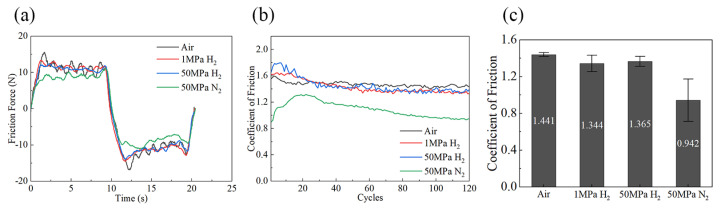
(**a**) Friction force–time curves in the 120th cycle for NBR under different atmospheres and pressures; (**b**) COF as a function of cycle number under different atmospheres and pressures; (**c**) Bar chart of COF for NBR under different atmospheres and pressures.

Ball-on-disk reciprocating friction and wear tests were conducted in ambient air, 1 MPa hydrogen (H_2_), 50 MPa H_2_, and 50 MPa nitrogen (N_2_). During testing, the friction force exhibited a periodic variation with the reciprocating cycles. Figure 4 shows the friction force–time curves recorded in the 120th cycle (i.e., the last cycle of the test, when the friction force had approached a steady state) under the four conditions. Within a single cycle, the friction force varied periodically: it first rose rapidly to a quasi-steady level and then fluctuated slightly around this value; when the sliding direction reversed, the friction force dropped abruptly into the negative range and then showed a similarly stable fluctuation in the opposite direction, forming a repeating pattern of “rise–stable fluctuation–drop to negative–stable fluctuation”. This behavior indicates a typical stick–slip motion at the steel ball/rubber interface.

The COF was averaged over the last 10 cycles to obtain the steady-state COF for each condition. Overall, the COF under all conditions evolved from an initial running-in stage to a stable stage. The results show that the 50 MPa N_2_ condition differs markedly from the other three, whereas the COF values in 1 MPa H_2_, 50 MPa H_2_, and ambient air are relatively close. Specifically, the COF–cycle curves in 1 MPa H_2_ and 50 MPa H_2_ exhibit highly consistent trends, with steady-state COFs of 1.344 ± 0.090 and 1.365 ± 0.054, respectively. The COF curve in ambient air is also similar to those in H_2_, but the stable-stage COF is slightly higher (1.441 ± 0.021), which is approximately 7.2% higher than that in 1 MPa H_2_. In sharp contrast, the 50 MPa N_2_ condition shows a substantially lower COF throughout the entire test; the steady-state COF is only 0.942 ± 0.230, which is about 31% lower than that in H_2_ at the same pressure. This pronounced difference demonstrates that the gas species plays an important role in governing the friction behavior of the NBR specimen.

**Figure 5:**
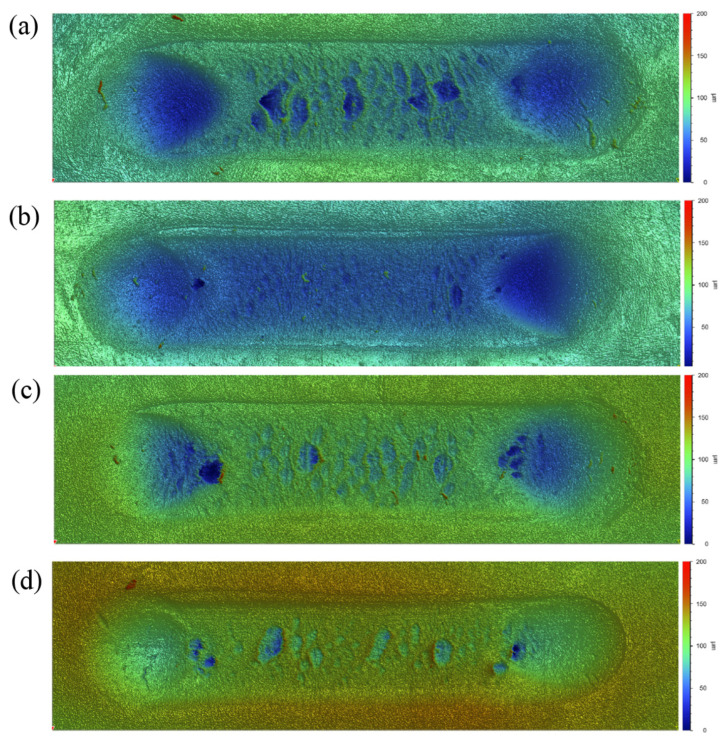
Three-dimensional surface topography of NBR wear tracks: (**a**) air, (**b**) 1 MPa H_2_, (**c**) 50 MPa H_2_, and (**d**) 50 MPa N_2_.

In terms of wear, the wear volume and wear scar width were calculated from three-dimensional (3D) surface topography after testing. The 3D morphologies of the wear tracks on NBR under the four dry-friction conditions are shown in Figure 5. A common feature is that the central region of the wear track exhibits pronounced wear patterns and tearing damage, whereas conspicuous pits are observed at both ends of the track. This difference originates from the change in motion state: in the central region the ball slides at a constant speed and sliding friction dominates, leading to wear patterns associated with stick–slip; at the ends, where the direction changes, the relative dwell time of the ball increases, and the prolonged action of the normal load results in permanent plastic deformation, forming pits.

**Figure 6:**
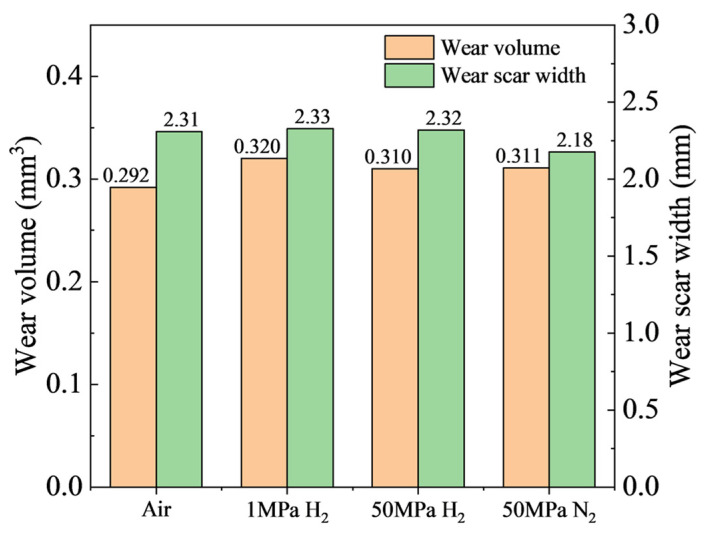
Wear volume and wear scar width of NBR under different atmospheres and pressures.

The wear volume and wear scar width of NBR under different atmospheres and pressures derived from the 3D topography are summarized in Figure 6. In terms of wear volume, the values are relatively close under the four conditions, ranging from 0.292 mm^3^ to 0.320 mm^3^. The largest wear volume is obtained in 1 MPa H_2_ (0.320 mm^3^), whereas the smallest is in ambient air (0.292 mm^3^). The wear volumes in 50 MPa H_2_ and 50 MPa N_2_ are nearly identical (0.310 mm^3^ and 0.311 mm^3^, respectively). Further analysis using the wear scar width shows that the widths in ambient air, 1 MPa H_2_, and 50 MPa H_2_ are similar (about 2.31–2.33 mm), whereas the 50 MPa N_2_ condition gives the narrowest wear scar (2.18 mm), consistent with the trend in COF. Compared with the N_2_ condition, the larger wear in 50 MPa high-pressure H_2_ can be attributed to two factors: (i) swelling of the rubber in high-pressure H_2_ reduces the hardness of NBR, thereby increasing the contact area between the ball and the rubber surface under the same normal load; and (ii) hydrogen uptake and swelling reduce the material strength, making it more susceptible to damage during wear. These effects jointly promote an increase in wear.

**Figure 7:**
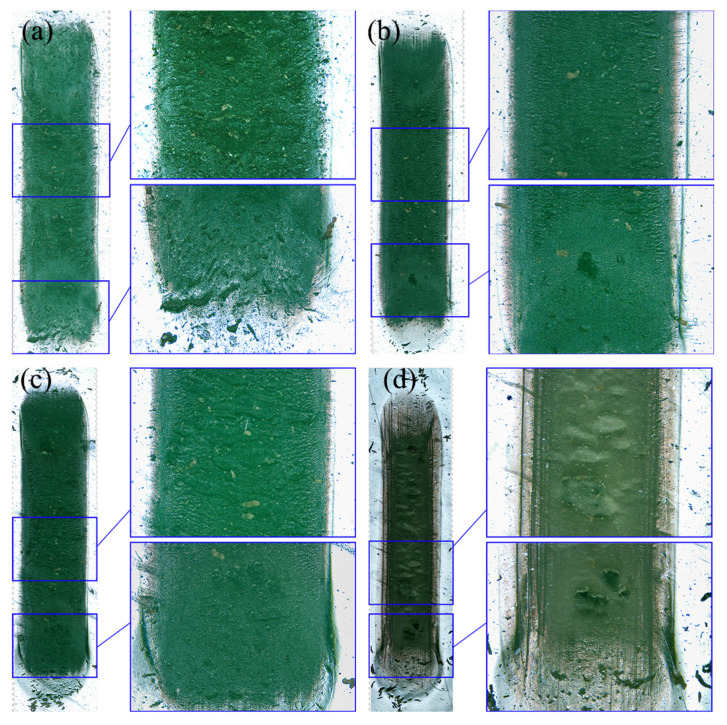
Optical micrographs of NBR wear tracks: (**a**) air, (**b**) 1 MPa H_2_, (**c**) 50 MPa H_2_, and (**d**) 50 MPa N_2_.

Optical microscopy observations (Figure 7) indicate that, under all four dry-friction conditions, the central region of the wear track shows evident wear patterns perpendicular to the sliding direction, together with some large pits formed by material tearing. At both ends, no obvious wear patterns are observed; instead, small pits caused by local material spalling are present. Wear debris can also be seen on the worn surface and outside the wear track. In ambient air, both the wear patterns and tearing traces are pronounced. In comparison, the damage in 1 MPa H_2_ is noticeably smaller, with shallower wear patterns and fewer tearing traces. When the H_2_ pressure increases to 50 MPa, the wear pattern size becomes larger and the degree of tearing increases. The wear morphology in 50 MPa N_2_ differs significantly, showing numerous pits formed by material spalling on the worn surface.

**Figure 8:**
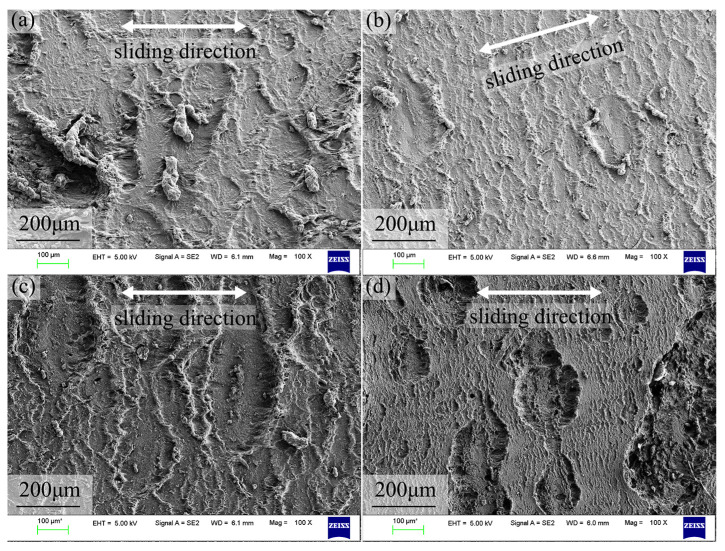
SEM images of NBR worn surfaces: (**a**) air, (**b**) 1 MPa H_2_, (**c**) 50 MPa H_2_, and (**d**) 50 MPa N_2_.

SEM observations show that periodic ridge-like wear patterns perpendicular to the sliding direction form on the worn surface in both air and H_2_, which are characteristic of worn rubber. In ambient air, in addition to the typical patterns, elongated debris and pits produced by material tearing are observed, indicating that adhesive wear dominates in air (Figure 8a). Figure 8b shows the worn morphology in 1 MPa H_2_, where the ridge height and spacing are significantly smaller than those in air; the surface undulation is clearly reduced, and no obvious tearing pits are observed. When the H_2_ pressure increases to 50 MPa, the ridge size and height increase, the surface roughness rises, and more fine particulate debris and local tearing traces appear, indicating that swelling-induced softening and strength reduction in high-pressure H_2_ aggravate adhesive wear of NBR. In 50 MPa N_2_ (Figure 8d), the worn morphology exhibits a non-uniform “dual” character: in some regions, lamellar material spalling occurs, forming irregular shallow pits; in other regions, relatively fine wear patterns remain, and the overall wear is mild without widespread surface damage. This suggests that, under 50 MPa N_2_, the NBR may still be in a running-in stage and has not yet developed a uniform wear surface; increasing the number of cycles may be required for the wear patterns to fully develop and for the worn morphology to become comparable to that under the other three conditions. Compared with air and 50 MPa H_2_, the overall wear severity of NBR is lower in 50 MPa N_2_.

**Figure 9:**
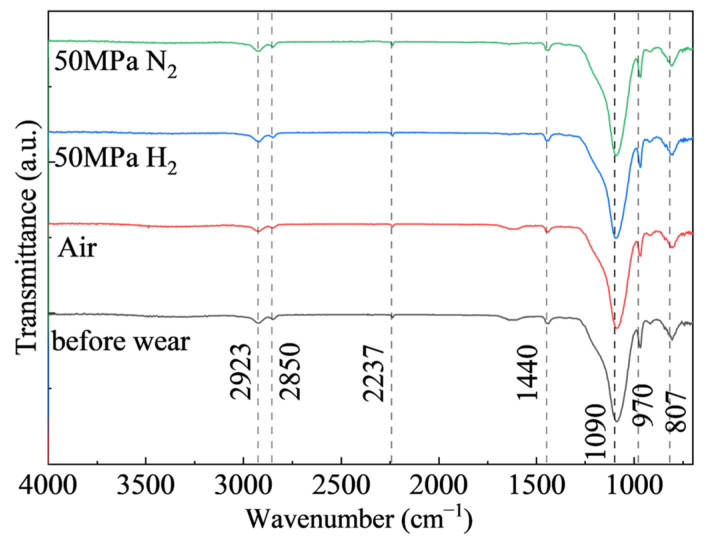
ATR-FTIR spectra of NBR surfaces before and after wear.

To examine whether tribochemical reactions occurred during wear, ATR-FTIR spectra were collected for the unworn specimen and selected worn specimens. Figure 9 shows the ATR-FTIR spectra of NBR in the unworn state and after wear in air, 50 MPa H_2_, and 50 MPa N_2_. No peak shifts or significant changes in relative peak intensity are observed; therefore, no obvious chemical reactions occurred during the friction and wear process.

### 3.2. Friction and Wear Behavior of NBR Under Lubricated Conditions

Compared with the severe fluctuations and high COF under dry friction, the friction curves under lubrication are much smoother, and the COF values are relatively low. In air and 50 MPa H_2_, the steady-state COFs are only 0.099 ± 0.006 and 0.105 ± 0.002, respectively, showing no significant difference. Relative to the dry-friction conditions (COF ≈ 1.3–1.4), the COF is reduced by approximately one order of magnitude. This indicates that the lubricant forms an effective lubricating film at the interface, which completely separates the NBR from direct contact with the steel ball, eliminates stick–slip between the ball and the rubber, and causes the friction behavior to be governed primarily by the internal shear of the lubricant itself, thereby reducing the influence of rubber material properties and the gas environment.

**Figure 10:**
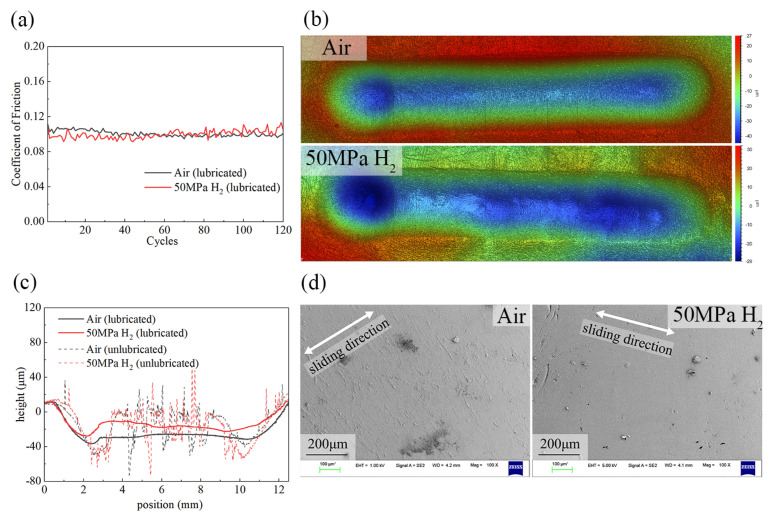
(**a**) COF versus cycle number under lubrication; (**b**) 3D surface topography of NBR wear tracks under lubrication: air (up) and 50 MPa H_2_ (down); (**c**) cross-sectional profiles of wear tracks under lubricated and dry conditions; (**d**) SEM images of NBR wear tracks under lubrication: air (**left**) and 50 MPa H_2_ (**right**).

Figure 10b shows the 3D morphology of the worn surface under lubrication. The wear track appears as a smooth and regular indentation, without any wear patterns or tearing damage. From the 3D topography, a centerline profile along the sliding direction shows a smooth concave shape under lubrication, indicating that adhesion is suppressed and the wear track geometry is mainly determined by the compressive indentation of the ball. The slightly deeper depression at the far left is attributed to the ball dwelling there before the test started, resulting in greater plastic deformation than in other regions. In contrast, the dry-friction profiles show pronounced serrated fluctuations, corresponding to ridge-like wear patterns and tearing pits. Notably, under dry friction, both ends of the wear track exhibit clear depressions because the ball dwells longer during reversal, whereas the average height of the central sliding region is higher than that under lubrication due to adhesive stretching of the rubber by the ball.

SEM further confirms that under lubrication, the NBR surface shows slight running marks, with no evidence of material fracture. In air, very mild wear marks can be observed, whereas in 50 MPa H_2_, almost no surface wear marks are visible. This indicates that the wear mechanism becomes mild abrasive wear or fatigue wear, with the wear severity suppressed to a very low level. Overall, these results demonstrate that the lubricating film effectively inhibits stick–slip-induced crack propagation and large-scale tearing damage, shifts the dominant wear mechanism from adhesive tearing to mild abrasive/fatigue wear, and thereby enables low-friction and low-wear performance in high-pressure hydrogen environments.

## 4. Discussion

Under dry friction, the interaction between the steel ball and the NBR material shows a specific pattern during each back-and-forth cycle. The friction force quickly increases, stabilizes for a while, and then suddenly changes direction when reversing—this stable fluctuation goes the other way. This sign reversal of the friction force corresponds to the load cell transitioning between tension and compression as the sliding direction reverses. The presence of fluctuations during the stable phase is a clear sign of stick-slip behavior, meaning that the way the surfaces stick together and slip apart plays a key role in how much friction is seen. Among the four atmospheres, the steady-state COFs in air, 1 MPa H_2_, and 50 MPa H_2_ are 1.441, 1.344, and 1.365, respectively, whereas the COF in 50 MPa N_2_ is significantly lower (0.942). This difference implies that the gas species is more influential than increasing the hydrogen pressure from 1 MPa to 50 MPa in terms of friction control.

High-pressure gas compression can densify the near-surface layer of rubber, thereby reducing friction. However, because hydrogen molecules are small and readily dissolve and diffuse into elastomers [23,24], H_2_ can induce swelling and reduce hardness and strength, thereby increasing the contact area, enhancing interfacial adhesion, and promoting tearing damage [25]. By contrast, nitrogen molecules are larger and have weaker swelling effects; therefore, at the same pressure, N_2_ exhibits lower friction. In addition, the relative humidity in air tests is 40–60%, while the high-purity H_2_/N_2_ atmospheres are much drier. Moisture may provide a lubricating effect within a certain range, but previous studies suggest that humidity can also significantly increase adhesion [26,27], which could contribute to the slightly higher friction in air compared with high-purity gas environments. The chemical state of the metallic counterface should also be considered. It has been reported that surface oxides on stainless steel can be reduced in high-pressure hydrogen environments [28]. This exposure of a more active metal surface may enhance metal/rubber adhesion, thereby promoting adhesive–tearing wear. Therefore, at 50 MPa H_2_, two competing effects may coexist: pressure-induced densification, tending to reduce friction, and hydrogen-induced swelling/metal-surface reduction, tending to increase adhesion, leading to a COF close to that in 1 MPa H_2_.

In terms of wear, the wear volumes under the four dry-friction conditions fall within 0.292–0.320 mm^3^, whereas the wear scar width better reflects the expansion of the contact region: the widths are about 2.31–2.33 mm in air and H_2_, but about 2.18 mm in 50 MPa N_2_. This is because, under coupled normal and tangential loading, rubber undergoes irreversible permanent deformation; thus, the volume loss calculated by integrating 3D profiles includes two contributions: true material removal and permanent plastic deformation, which may mask the real differences in wear across different gas environments. In contrast, wear scar width is more sensitive to changes in contact area and can indirectly capture the tendency toward softening, adhesion enhancement, and contact expansion. Meanwhile, ATR-FTIR shows no new absorption peaks or peak shifts, indicating that under the present conditions (50 MPa, 6 h hydrogen exposure), the degradation is dominated by physical swelling and microstructural damage rather than chemical bond scission or hydrogenation reactions. This supports the interpretation that friction and wear differences are driven mainly by changes in mechanical state and interfacial adhesion.

After grease coating, the steady-state COFs in air and 50 MPa H_2_ decrease to 0.099 and 0.105, respectively, and the fluctuations are greatly reduced. Although the lubricant may be partially displaced during the sliding process, the sustained low friction implies that a functional film is maintained. This indicates that a stable lubricating film effectively separates direct metal–rubber contact and fundamentally suppresses the stick–slip contribution to friction. Furthermore, the steady friction trend suggests that the grease maintained its structural integrity sufficiently to prevent lubrication failure during the test. Moreover, the PTFE-based grease can form a relatively stable continuous film in high-pressure gas environments, thereby diminishing the effect of gas-species-dependent adhesion and counterface chemical state. Morphological characterization shows that the wear track changes from ridge-like patterns, tearing pits, and debris accumulation under dry friction to a smooth and regular indentation under lubrication; both 3D profiles and SEM reveal slight damage. From an engineering perspective, establishing a stable lubricating film using an appropriate grease in high-pressure hydrogen sealing systems can reduce friction by approximately one order of magnitude, markedly mitigate surface damage, and potentially improve the long-term reliability of sealing pairs by lowering surface roughness and reducing the probability of tearing pits/leakage path formation.

It should be noted that the reciprocating test parameters in this study follow the standard-referenced setting (normal load 8.2 N, stroke 10 mm, speed 1 mm/s, 120 cycles) at room temperature, and the friction and wear tests were performed in situ in 50 MPa hydrogen. Future work incorporating pressure cycling closer to real sealing conditions, coupled temperature variations, long-duration wear, variable loads, and systematic evaluation of grease aging/compatibility would help clarify the relative roles of hydrogen-induced softening defect evolution, adhesive tearing, and lubricating-film stability in service-life prediction, and provide a more robust basis for selecting sealing materials and lubrication strategies for high-pressure hydrogen applications.

## 5. Conclusions

In the reciprocating ball-on-disk configuration (316L stainless-steel ball against NBR), the stable-stage COF values in ambient air, 1 MPa H_2_, and 50 MPa H_2_ are 1.441, 1.344, and 1.365, respectively, whereas the COF in 50 MPa N_2_ decreases to 0.942, indicating a pronounced influence of gas medium on NBR adhesion and friction behavior. The wear volumes under the four dry conditions are close (0.292–0.320 mm^3^), but the wear-track width in 50 MPa N_2_ is smaller (2.18 mm) than those in air and hydrogen, consistent with its lower friction. After grease coating, the steady-stage COF in ambient air and 50 MPa H_2_ decreases to 0.099 and 0.105, and the wear morphology transforms from ridge-like patterns/tear pits to regular and smooth indentations with only mild running marks, demonstrating that a lubricating film can effectively suppress stick–slip and adhesive wear and enable a stable low-friction, low-wear interface in high-pressure hydrogen.

## Figures and Tables

**Table 1:** Key formulation information of the specimen material.

Reagent	Type	Loading (phr)	Manufacturer
Nitrile butadiene rubber (NBR)	Raw rubber	100	Shanghai Duokang Industrial Co., Ltd., Shanghai, China
Sulfur	Vulcanizing agent	2	Zhejiang Yongjia Chemical Co., Ltd., Wenzhou, China
Stearic acid (SA)	Vulcanization activator	2	Sinopharm Chemical Reagent Co., Ltd., Shanghai, China
Zinc oxide (ZnO)	Vulcanization activator	2	Aladdin Reagent Co., Ltd., Shanghai, China
N-cyclohexyl-2-benzothiazolesulfenamide (CZ)	Vulcanization accelerator	1	TCI (Shanghai) Development Co., Ltd., Shanghai, China
N-(1,3-dimethylbutyl)-N′-phenyl-p-phenylenediamine (6PPD)	Antioxidant (anti-aging agent)	1	Kunshan Anzhe Chemical Co., Ltd., Suzhou, China
Silicon dioxide (SiO_2_)	Filler	60	Evonik Degussa Co., Akron, OH, USA

**Table 2:** Main technical specifications of the test rig.

Item	Specification
Maximum working pressure (MPa)	140
Test temperature range (°C)	−60 to 200
Normal load (N)	Max ≥ 20, adjustable
Stroke (mm)	Max ≥ 20, adjustable
Sliding speed (mm/s)	Max ≥ 50, adjustable

**Table 3:** Summary of the test matrix for tribological evaluation.

Test Condition	Gas	Pressure (MPa)	Lubrication State	Number of Repeats
Ambient Air	Air	Ambient	Unlubricated	3
1 MPa H_2_	H_2_	1	Unlubricated	3
50 MPa H_2_	H_2_	50	Unlubricated	3
50 MPa N_2_	N_2_	50	Unlubricated	3
Ambient Air (Lubricated)	Air	Ambient	Lubricated	3
50 MPa H_2_ (Lubricated)	H_2_	50	Lubricated	3

## Data Availability

All data generated or analyzed during this study are included in the article. Further inquiries can be directed to the corresponding authors.

## Abbreviations

The following abbreviations are used in this manuscript:

H_2_	Hydrogen
N_2_	Nitrogen
NBR	Nitrile butadiene rubber
SEM	Scanning electron microscopy
ATR-FTIR	Attenuated total reflectance Fourier transform infrared spectroscopy
VMQ	Silicone rubber
FKM	Fluoroelastomer
EPDM	Ethylene propylene diene monomer
COF	Coefficient of friction
